# 

**Published:** 1888-12

**Authors:** 


### TABLE OF CONTENTS.

ORIGINAL AND SELECTED ARTICLES.

Vomiting and its treatment, by R. C. Word, M. D., Professor of Physiology in the South ern Medical College. Atlanta, Ga ..... 441
A case of shoulder presentation, by J. C. Beau champ. M. D., of Ga. ..... 446
The modern treatment of neuralgia, by L C. Gray, M. D, Professor of Mental and Nerv ous Diseases in the New York Polyclinic ..... 447
Treatment of dysmenorrhœa by mental suggestion or hypnotism, by Arthur C. Hugenschmidt, M. D.,(Univ. of Pa,) Paris, France.. 453
Ovaries vs. testicles, by Edward Borck, A. M., M. D, St. Louis, Mo ..... 455
Diarrhœa ..... 457

ABSTRACTS AND GLEANINGS.

Shock ..... 458
The Turkish bath ..... 459
The German treatment of obesity ..... 460
Forty-three gall stones passed by a child two and a half years old—recovery ..... 461
Hot air inhalations for phthisis ..... 462
Monthly summary of medical progress ..... 463
Antipyrin in pertussis ..... 46[illegible]
Corrosive sublimate as a practical disinfectant; lipanine ..... 466
Headache; catarrh cures ..... 467
The venom of the rattlesnake and the gila monster; collinsonia ..... 468
Report to the Congress for the study of tuberculosis; the treatment of ulcers; the purgative action of glycerin enemata ..... 469
Suture of a wound of the liver; iodoform and tar; canadol as an anæsthetic ..... 470

SCIENTIFIC ITEMS.

The anæsthetic revelation; causes of fire ..... 471
A toad in solid coal; the longest tangent in the world ..... 472

PRACTICAL NOTES AND FORMULÆ.

Diarrhœa; treatment of comedones; substitute for cod-liver oil ..... 473
Pomade against neuralgia; tetanus: epilepsy; Whooping-cough; pleurisy; pneumonia ..... 474

EDITORIALS AND MISCELLANEOUS.

Our journal; don't stop your journal; hypnotism ..... 475
Lawless liberty ..... 476
Editorial brevities.
Books and pamphlets received ..... 479
Special notes ..... 48[illegible]

